# Women empowerment and access to maternity and reproductive healthcare in Pakistan: cross-validation of a Survey-based Index in Afghanistan (SWEI-A)

**DOI:** 10.1186/s12905-022-02031-2

**Published:** 2022-11-16

**Authors:** Omid Dadras, Mohammadyasin Dadras, Leila Jafari, Takeo Nakayama, Fateme Dadras

**Affiliations:** 1 Department of Global Public Health and Primary Care, University of Bergen (UiB), Bergen, Norway; 2 Section Global Health and Rehabilitation, Western Norway University of Applied Sciences, Bergen, Norway; 3 School of Nursing, Zahedan University of Medical Sciences, Zahedan, Iran; 4 Department of Public Health and Nursing, School of Medicine and Health Sciences, Norwegian University of Science and Technology (NTNU), Trondheim, Norway; 5 Department of Health Informatics, School of Public Health, Kyoto University, Kyoto, Japan; 6 Department of Gynecology and Obstetrics, Graduate School of Medicine, Tehran University of Medical Sciences, Tehran, Iran

**Keywords:** Women empowerment, Pakistan, Index, Reproductive health, Maternity care

## Abstract

**Background:**

Despite the obvious violation of women’s rights in Pakistan and the vital necessity for women empowerment, a unified country-specific index measuring women empowerment is not yet available. This study cross-validated a survey-based women empowerment index from Afghanistan to be used in Pakistan.

**Methods:**

The data for married Pakistani women aged 15–49 in the 2017–18 Pakistan demographic health survey was used to construct the final model using the explanatory and confirmatory factor analyses. The Cronbach’s alpha test examined the internal consistency of the developed index. To assess the convergence validity of the index, the association of each emerged domain with indicators of access to reproductive and maternity care was assessed by Poisson regression analysis adjusting for wealth index**.**

**Results:**

The final index had six domains; namely, labor force participation, attitude toward violence, decision-making, access to healthcare, literacy, age at critical life events predicting women empowerment of married Pakistani women with decent reliability (Cronbach’s α = 0.70), and validity (SRSEA&SRMR < 0.05, CFI&TLI > 0.92). The emerged domains were significantly associated with at least one of four indicators for access to reproductive and maternity care; indicative of a favorable convergence validity.

**Conclusion:**

Pakistan and Afghanistan are associated as brother countries with shared religious and ethnocultural identities in which women are perceived inferior to men and in critical need of empowering efforts. The results of this study reflect upon this resemblance in sociocultural structure by yielding similar domains for women's empowerment in Pakistan building upon an index previously developed for Afghan women. The developed index could inform the design of future policies, interventions, and research recognizing the important indicators of women empowerment in Pakistan and could enhance the comparability of the results across future studies.

**Supplementary Information:**

The online version contains supplementary material available at 10.1186/s12905-022-02031-2.

## Introduction

Gender equality and women empowerment are the essence of the Sustainable Development Goal (SDG) five and are vital to global human development. Empowerment is defined as enabling underprivileged populations by removing the existing barriers toward individual decision-making and autonomous action that can enhance the individuals’ well-being [1, 2]. For the last two decades, enormous global efforts have been engaged by several parties to improve gender equality by offering equal rights and opportunities for education, health care, and occupation for both males and females [3]. To achieve this, however, women empowerment is essential through three main streams including *Agency* indicating the decision-making abilities regardless of the existing power structure; *Resources* that are described as channels through which one exercises agency such as education, health, and physical assets; and *Achievements* that are the product of agency such as economic and socio-political gains [2].

Although gender-based issues are widespread global concerns, in some poor-resourced countries such as Pakistan and Afghanistan, these issues turned into a humanitarian crisis and have undermined the current efforts to achieve equal shares for both genders in the country’s socioeconomic development [4, 5]. In both Pakistan and Afghanistan, the culture and gender-based norms introduce a huge gap in order and hierarchy of society that recognize women inferior to men with less power and authority in decision-making [5–7]. In fact, the poor performance of Pakistan and Afghanistan concerning gender equality efforts placed them at the end of the list, respectively at 145 and 146 ranks, in the latest Gender Gap Index 2022 [22]. Several studies have shown that women's empowerment benefits both women as individuals and society as a collective and can contribute to the development of the whole society [6–9]. It has been linked to improved men's and women’s health [10], reduced child mortality and morbidity [11], enhanced use of modern contraception, adequate antenatal care (ANC), institutional delivery, and skilled birth attendance [9, 12, 13]. Besides, it has been shown that the children of empowered women are less likely to suffer from malnutrition and their daughters are more likely to spend longer time in education and receive equal treatment as their sons in inheritance [14].

The multidimensionality of women empowerment has caused challenges in the measurement and comparability of the results across different contexts [15, 16]. Several scales have been developed to measure women's empowerment such as the Gender-based Development Index (GDI), the Gender-based Empowerment Measure (GEM), and the Gender-Equality Index (GEI) in which composite indices estimate the gender-based disparities in terms of basic capabilities of male and female; nonetheless, there are some methodological shortcomings such as the relevance and importance of data and geographical coverage that limited the use of such indices [17]. In addition, the choice of indicators is often limited by what is available at the national level and considered a disadvantage in poor-resourced countries such as Afghanistan and Pakistan where the existent indicators are not truly representative of gender-based disparities [18]. Therefore, to capture the multidimensional structure of women’s empowerment in a specific context, it is necessary to define reliable and context-specific variables. This would assist future research and policy to measure women’s empowerment using a unified scale and facilitate the periodical surveillance and progress assessment of what has been achieved.

Against this backdrop, we developed a survey-based country-specific index; namely, survey-based women empowerment index in Afghanistan (SWEI-A) [4] that demonstrated promising structural validity and internal consistency in measuring empowerment among married women aged 15–49 years in Afghanistan using the relevant indicators from the 2015 Afghanistan Demographic Health Survey (ADHS). Since Pakistan and Afghanistan‒two neighboring countries‒ share ethnocultural roots that impact women empowerment; in this study, we aimed to cross-validate the survey-based women empowerment index in Afghanistan (SWEI-A) to be used for Pakistani women drawing upon the existent indicators that have been collected for women aged 15–49 years in the Pakistan DHS 2017–18. We also assessed the convergence validity of the modified index by examining the association between four indicators of access to reproductive and maternity care and emerged domains in factor analyses. It has been well-documented that there is a strong association between these indicators and women empowerment [19, 20]. Previous studies in Pakistan have failed to use a unified index to measure women's empowerment [21–24] and thus the results are often inconsistent across different settings. For all we know, this is the first country-specific survey-based index that has been developed to measure women's empowerment among married Pakistani women aged 15–49 and could have significant policy implications and enhance the comparability of the results across future studies in Pakistan.

## Methods

### Study setting

This study used cross-sectional data from the 2017–18 Pakistan Demographic Health Survey (PDHS 2017–18). PDHS 2017–18 is the latest nationally representative survey conducted by the Pakistan National Institute of Population Studies and the ICF International funded by the United States Agency for International Development (USAID).

### Study design and population

PDHS 2017–18 collected data for women aged 15–49 years and their children under 5 years old through a stratified two-stage cluster sampling to estimate the key indicators at the national level, in urban and rural areas. The survey was conducted across four provinces (Punjab, Sindh, Khyber Pakhtunkhwa, Balochistan); Azad Jammu & Kashmir (AJK) and Gilgit Baltistan (GB); Islamabad Capital Territory (ICT); and the former Federally Administrated Tribal Areas (FATA) in Pakistan. In the first stage, 580 clusters (enumeration areas from the previous national census consist 200–250 households) were selected. In the second stage, through an equal probability systematic selection process, 16,240 households were selected within 580 clusters. To obtain representative estimates at the national level, sampling weights were calculated and applied. A sample of the women aged 15–49 years (*n* = 15,930) who were either permanent residents of the selected households or visitors who stayed in the households the night before the survey were recruited after informed consent, out of them 15,068 women were successfully interviewed in PDHS, and their response rate was 94.6%. More details could be found in the PDHS 2017–18 final report [25]. For the purpose of the present study, the analysis was restricted to married women aged 15–49 years old (*n* = 14,502), because for some variables the data were only collected for married women.

### Study variables

## Variable selected for the explanatory (EFA) and confirmatory factor analysis (CFA)

A total of 26 important variables related to women empowerment [15, 16, 20, 26–29] including those from our previous study [4] that were available in 2017–18 PDHS, were selected for EFA CFA. All categorical variables were either recoded or used in their original format based on their suggested direction and influence on women empowerment so that the categories with higher ranks represent higher levels of empowerment and those with lower ranks indicate low empowerment [16, 28]. A summary of 26 variables, eight domains, and four dimensions that were conceptualized in this study along with the details of recoded variables, their frequency, and distribution were provided in supplementary materials (Table S1, and S2).

### Economic dimension

This dimension included two domains; namely, *labor force participation* and *property-owning*. *Labor force participation* included the following indicators: respondent's occupation, type of earning from respondent's work, seasonality of respondent's occupation, income ratio (women/men), and work autonomy. *Property owning* was represented by legally owning a house or land variables.

### Socio-cultural dimension

This dimension included three domains; *decision-making*, *attitude toward violence*, and *age at critical life events*. Participation in *decision-making* was assessed by three items, namely: (1) person who decides respondent's healthcare; (2) person who decides on large household purchases; and (3) person who decides whether the respondent can visit her family or relatives. *Attitudes toward violence* were assessed using five variables describing whether beating was justified if the wife: goes out without telling her husband; neglects the children; argues with her husband; refuses sex with her husband; burns food. *Age at critical life events* domain was measured by two indicators including age at first birth and age at first cohabitation [20].

### Education dimension

This dimension included one domain; namely, *literacy* which was measured by the ability of the participants to read and the highest education level of participants.

### Health dimension

This dimension includes *negotiating sex* and *access to healthcare* domains. Women's ability to negotiate sex was measured by indicators describing if they could refuse sex or ask their partner to use a condom. *Access to healthcare* was classified by four indicators examining the difficulty in getting medical help (not a big problem = 1, big problem = 0), namely: (1) receiving permission before getting medical help; (2) having money for healthcare; (3) distance to health facility; (4) not wanting to go healthcare facility alone [29].

## Variables related to access to reproductive and maternity care

Four indicators related to access to reproductive and maternity care were selected as outcome variables including, 1) unmet needs for family planning, 2) adequate ANC, 3) institutional delivery, and 4) skilled birth attendance.

- a) Unmet needs for family planning: Unmet need was defined as the unmet need for limiting (i.e. women whose most recent pregnancy was not wanted at all, fecund women who did not use contraception despite their desire to have no more children, women who were postpartum amenorrheic for 2 years following an unwanted birth and were not using contraception) and spacing (i.e. women whose most recent pregnancy was not wanted initially but wanted later, fecund women not using contraception who were undecided when/if they wanted a to have a child or who wanted a child 2 + years later, and women who were postpartum amenorrheic for 2 years following a mistimed birth and were not using contraception) [15]. The relevant questions had dichotomous response alternatives (i.e., ‘yes’ or ‘no’ responses) and unmet needs for family planning were coded as “yes = 1” and “no = 0”.
- b) Adequate ANC: Based on the World Health Organization (WHO) recommendation, having at least four ANC visits is necessary for optimal maternal and child outcomes [23]. Therefore, adequate ANC was coded as 'yes = 1' for women with at least four ANC visits before their most recent (4 + ANC visits) in the last five years and coded as 'no = 0' if there were fewer than four visits.
- c) Institutional delivery: This variable is coded into “yes = 1” indicating delivery at health facilities and “no = 0” indicating delivery at home/elsewhere.
- d) Skilled birth attendance: Defined as and coded “1” if birth is delivered with the assistance of a doctor, nurse, midwife, lady health visitor, or community midwife; otherwise, it was coded “0”.

### Data analysis

**Fig. 1:**
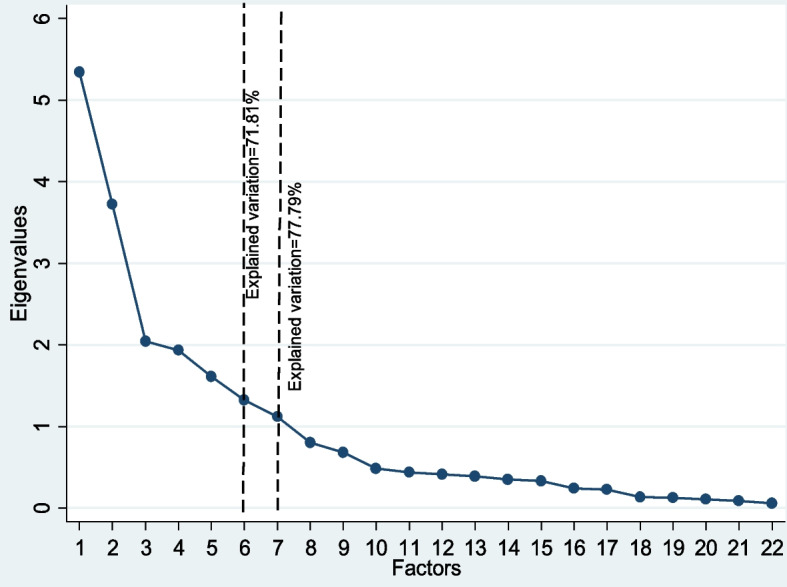
Scree plot of eigenvalues plotted against factors, including 26 variables used for EFA

Data analysis was conducted in four steps following the procedure from our previous paper [4]. First, the variables were extracted from PDHS 2017–18 dataset and either recoded or retained in their original forms for factor analysis (Table S1), then the dataset was randomly split into half using the STATA command “splitsample”. Assuming that homogenous samples of married women aged 15–49 years are being generated, the first half was used for EFA and the second half was later used for CFA to assess the construct validity as recommended in previous literature [30, 31]. The suitability of data for EFA was tested using the Kaiser–Meyer–Olkin (KMO) test of sampling adequacy and Bartlett test of sphericity [32] in which, respectively, values greater than 0.70 and *p*-value < 0.05 are considered favorable. In the second step, the first half of the sample was used to identify the latent constructs that reflect women’s empowerment using EFA. The decision on which domains to be retained was made based on the eigenvalue (> 1), scree plot (Fig. 1), and the amount of explained variability by each individual domain. The variables with a loading factor < 0.3 and those loaded on more than one domain were dropped in the further analysis as recommended by Stevens 2009 [33]. To construct the final model and obtain the structural domains‒empowerment indices‒oblique rotation was adopted over orthogonal rotation to account for the potential correlation between factors [29]. In the third step, the internal reliability of the overall index and individual domain was assessed by Cronbach’s α test (Table 2) [34, 35] and domains with a Cronbach’s α value < 50% as well as the variables that removing them significantly improve the Cronbach’s α coefficients, were dropped [36, 37]. In the last step, the construct validity of the index was assessed by confirmatory factor analysis (CFA) in the other half of the sample to estimate how well the measured variables represent the number of emerged constructs. The CFA produces the fit statistics based on the covariate structure of observed data (Table 3) to determine the appropriateness of the model. These include the Root Mean Squared Error of approximation (RMSEA) which represent the parsimony of an index; the Comparative Fit Index (CFI), Tucker-Lewis index (TLI), and Standardized Root Mean Squared Residual (SRMR) which represent relative and absolute fit of the index [38]. An index with good construct validity has RMSR and RMSEA < 0.05 and CFI and TLI more than 0.95 [39]. In addition to CFA, to evaluate the convergent validity of the final model, the association between the emerged domains and four indicators of access to reproductive and maternity care; namely unmet needs for family planning, adequate ANC, institutional delivery, and skilled birth attendance were measured. Higher access to reproductive and maternity care services has been observed among more empowered women [9, 12, 19, 40, 41]. These associations were estimated using Poisson regression as recommended by Barros et al. [42] and adjusted for household wealth to assess the association of empowerment with the four outcomes of interest independent from the household’s wealth [20]. The categories (low, medium, high) for women empowerment domains were obtained by pooling the individual indicators’ scores and approximating the terciles as the cutoff points [20, 43] and the women in high tercile was compared to the women low tercile (reference group) in terms of the utilization of reproductive and maternity care services to avoid masking the effect of women empowerment by the scores in the middle tercile and highlighting the significant association between each construct and the outcome as suggested by previous literature (Table 4) [20]. All the analyses were performed in STATA software version 16 and the p-value < 0.05 was considered a significant statistical level. In both EFA and CFA analysis, the missing data were treated using the listwise deletion approach, assuming that the data were missing completely at random (MCAR). MCAR means that the probability of obtaining a particular pattern of missing data is not dependent on the values that are missing and when the probability of obtaining the missing data pattern in the sample is not dependent on the observed data [44]. Although this approach has some limitations and the assumptions may not completely hold, the literature has shown that when the data are MCAR there is little difference in the estimation bias for listwise deletion, pairwise deletion, and maximum likelihood in structural equation modeling [45].

## Results

### Validity and reliability of the survey-based women empowerment index

A total of 14,502 married Pakistani women aged 15–49 years were included in this study. The sample was divided into half; the first half including 7257 married women was included in the EFA to explore the latent factors and the other half including 7245 women were included in the CFA to examine the construct validity of the index. Aside from the correlation matrix that indicated an acceptable level of correlation, the value for the KMO measure of sampling adequacy was 0.82, and the Bartlett test of sphericity was significant at a p-value < 0.001; indicating the suitability of data for EFA. The initial EFA model included 26 variables; however, the four variables including the “can ask the partner to use condom”, and “can say no to sex” in the *access to healthcare* domain, “income ratio” in *labor force participation* domain, and “frequency of reading newspaper” in *literacy* domain were dropped in further analysis either due to significantly different loading on one factor as compared to other variables or due to overlap with other variables loaded on different factors. The final model included 22 variables loaded on seven factors with eigenvalues > 1 (1.32–3.77) and explained 77.79% of the variation in the data. The first (17.13%) and second (16.87%) factors/domains, indicating *labor force participation* and *attitude toward violence*, accounted for the biggest portion of variation explained by the final model. Other factors/domains including *access to healthcare*, *decision-making*, *literacy*, and *property-owning* contributed to 10.53%, 10.26%, 8.52%, 8.51%, and 5.98% of the total explained variation by 7-factor model (Table 1). The internal consistency of the model was assessed by Cronbach’s alpha coefficients across 22 indicators and seven domains in the final model. As can be seen in Table 2, the value of Cronbach’s alpha coefficient was equal to or more than 0.70 for individual indicators and domains except for property-owning; therefore, we excluded this domain from the final model. The Cronbach’s alpha coefficient for the whole index was almost 0.70 and did not change significantly after excluding the *property-owning* domain; indicating an acceptable level of internal consistency in both the 6-factor and 7-factor models. In the end, the construct validity of the 6-factor and 7-factor models was assessed by CFA, taking into account the covariate components in the models using the structural equation modeling. The goodness-of-fit test indicated an acceptable level of fit with a Likelihood ratio, RMSEA, and SRMR ≤ 0.05 and CFI and TLI values > 0.95 for the 7-factor model but more significant indices for the 6-factor model; indicating a better fit for the 6-factor model (Table 3). Thus, the 6-factor model, excluding the *property-owning* domain was selected as the best and the final model.

**Table 1:** Factor loading values for individual variables and explained variation by each domain

Factor	Variables	loading	Variation (%)
^a^ F1	Occupation	0.97	17.13
	Earning	0.97	
	Work autonomy	0.95	
	Seasonality	0.96	
^b^ F2	Justified if goes out without telling husband	0.84	16.87
	Justified if neglects children	0.84	
	Justified if argues with husband	0.86	
	Justified if refuses sex	0.83	
	Justified if burns food	0.78	
^c^ F3	Permission	0.77	10.53
	Money	0.77	
	Distance	0.78	
	Going Alone	0.65	
^d^ F4	Women's health	0.84	10.26
	Large household purchases	0.86	
	Visiting relatives/family	0.83	
^e^ F5	Educational level	0.91	8.52
	Literacy	0.93	
^f^ F6	Age at cohabitation	0.93	8.51
	Age at first birth	0.95	
^g^ F7	House ownership	0.81	5.98
	Land ownership	0.81	

^a^ Factor/Domain 1: Labor Force Participation

^b^Factor/Domain 2: Attitudes towards violence

^c^Factor/Domain 3: Access to Healthcare

^d^Factor/Domain 4: Decision-making

^e^Factor/Domain 5: Literacy

^f^Factor/Domain 6: Age at critical Life events

^g^Factor/Domain 7: Property-owning

**Table 2:** The internal reliability of individual items, domains, and whole index

**Domain (score range)**	**Variables**	**Cronbach’s** α	
		**Item**	**Overall**
Labor Force Participation (0–13)	Occupation	0.94	0.96
	Earning	0.96	
	Work autonomy	0.96	
	Seasonality	0.94	
Attitudes towards violence (0–5)	Justified if goes out without telling husband	0.87	0.90
	Justified if neglects children	0.88	
	Justified if argues with husband	0.87	
	Justified if refuses sex	0.88	
	Justified if burns food	0.90	
Access to Healthcare (4–8)	Permission	0.72	0.78
	Money	0.72	
	Distance	0.69	
	Going Alone	0.76	
Decision-making (0–6)	Women's health	0.78	0.83
	Large household purchases	0.74	
	Visiting relatives/family	0.78	
Literacy (0–5)	Educational level	0.92	0.92
	Literacy	0.92	
Age at critical Life events (22–79)	Age at cohabitation	0.91	0.91
	Age at first birth	0.91	
Property-owning (0–6)	House ownership	0.47	0.47
	Land ownership	0.47	
Total index	Included Factor 7		0.699
	Without factor 7		0.702

**Table 3:** The goodness of fit tests for CFA; Construct validity

	**Likelihood ratio (*p*-value)**	**RMSEA**^**1**^	**CFI**^**2**^	**TLI**^**3**^	**SRMR**^**4**^
Model 1^a^	< 0.001	0.031	0.955	0.952	0.02
Model 2^b^	< 0.001	0.007	0.966	0.957	0.001

^1^*RMSEA* Root Mean Squared Error of Approximation

^2^*CFI* Comparative Fit Index

^3^*TLI* Tucker-Lewis index

^4^*SRMR* Standardized Root Mean Squared Residual, a Include factor 7 (property-owning), b Without factor 7 (property-owning)

### Convergence validity; the association between emerged domains and reproductive and maternity care access indicators

As Table 4 indicates, there was a significant association between at least one of the four indicators of reproductive and maternity care access and emerged domains in the final model (6-factor model), particularly *access to healthcare*, *literacy*, and *decision-making*. It appeared that the women who scored high in *access to healthcare* domain were more likely to have unmet needs for family planning (PRR = 1.29; 95%CI: 1.08–1.53), adequate ANC (PRR = 1.19; 95%CI: 1.08–1.30), institutional delivery (PRR = 1.12; 95%CI: 1.04–1.21), and skilled birth attendance (PRR = 1.14; 95%CI: 1.07–1.23) as compared to women with low scores in this domain after adjustment for wealth index. Likewise, the likelihood of having adequate ANC, institutional delivery, and skilled birth attendance were 19–54% higher among women with high levels of literacy and decision-making abilities in comparison to the women with low literacy and decision-making abilities. With regards to the *age at critical life events* domain, the women with high scores were more likely to have institutional delivery (PRR = 1.14; 95%CI: 1.03–1.26), and skilled birth attendance (PRR = 1.19; 95%CI: 1.08–1.32) compared to those with low scores. For *labor force participation* and *attitude toward violence* domains, the association with access to reproductive and maternity care indicators was weaker and only significant for unmet need for family planning (PRR = 0.82; 95%CI: 0.69–0.98) and adequate ANC (PRR = 1.27; 95%CI: 1.14–1.42), respectively.

**Table 4:** The association between emerged domains and four reproductive and health care access indicators among married women aged 15–49 years in Pakistan (PDHS 2017–18)

Dimensions	Domains	Unmet family planning	Adequate ANC	Institutional delivery	Skilled worker delivery
		**PRR (95%CI)**^**a**^	**PRR (95%CI)**^**a**^	**PRR (95%CI)**^**a**^	**PRR (95%CI)**^**a**^
Economic	Labor Force Participation ^b^	0.82 (0.69–0.98)*	1.09 (0.97–1.22)	0.96 (0.91–1.08)	1.02 (0.94–1.11)
Socio-cultural	Attitudes towards violence ^b^	0.90 (0.78–1.03)	1.27 (1.14–1.42)*	1.04 (0.97–1.11)	1.05 (0.98–1.13)
	Decision-making	0.96 (0.84–1.10)	1.24 (1.12–1.37)*	1.19 (1.10–1.29)*	1.19 (1.09–1.29)*
	Age at critical Life events^b^	0.95 (0.61–1.47)	1.19 (0.99–1.44)	1.14 (1.03–1.26)*	1.19 (1.08–1.32)*
Education	Literacy^b^	0.92 (0.78–1.08)	1.54 (1.40–1.69)*	1.22 (1.15–1.30)*	1.24 (1.16–1.32)*
Health	Access to Healthcare^b^	1.29 (1.08–1.53)*	1.19 (1.08–1.30)*	1.12 (1.04–1.21)*	1.14 (1.07–1.23)*

^*^p-value < 0.05

^a^*PRR (95% CI)* Prevalence rate ratio and 95% confidence interval, adjusted for the wealth index

^b^the high tertile was compared to the low tertile (reference group)

## Discussion

This study was a cross-validation of our previously developed index measuring women's empowerment in Afghanistan (SWEI-A) [4] to be used among married women aged 15–49 years in Pakistan. The original index was designed to measure women's empowerment in Afghanistan across seven domains; namely, *labor force participation, attitude toward violence, decision-making, access to healthcare, literacy, age at critical life events,* and *property-owning*; however, our analysis showed that the 6-factor model‒excluding the *property-owning*‒could better explain the woman empowerment among married women aged 15–49 years in Pakistan with an acceptable internal consistency (Cronbach’s α = 0.70) and construct validity (SRSEA&SRMR < 0.05, CFI&TLI > 0.95). To estimate the convergence validity of the developed index, we selected four indicators of access to reproductive and maternity care including the unmet need for family planning, adequate ANC, institutional delivery, and skilled birth attendance that have shown to be strongly associated with high women empowerment [9, 19, 20, 23, 46] and examine their associations with emerged domains. All six domains appeared to be strongly associated with at least one favorable outcome; indicative of a decent convergence validity of the 6-factor model. To ensure that the 6-factor model is superior to the 7-model factor in terms of convergence validity in the Pakistani female population, the association between property-owning and four favorable reproductive and maternity outcomes was examined which turn out to be insignificant.

Pakistan and Afghanistan are often associated as brother countries with deep historical ties, traditional resemblance, similar social composition, and shared religious and ethnocultural identities [47]. The violation of women’s rights has been a rife longstanding practice in both countries and rooted in the patriarchial man-dominant norms that place women in an underprivileged position in socio-cultural interaction creating an unbalanced dynamic that leaves women with lower autonomy and authority over critical decisions concerning their health and life. This could not only harm the health integrity of women but could also threaten the health of offspring [7, 43, 48]. Therefore, efforts to enhance women's empowerment at both individual and societal levels are essential to encourage women’s engagement in social, economic, and political interactions, reduce the existing gaps, and enhance the representativeness of women in society's socioeconomic development. However, one of the necessities to achieve this goal is to quantify women's empowerment across the different domains that have been suggested in previous studies [11, 20, 28, 29, 49]. The similarities in the socio-cultural composition of Afghanistan and Pakistan encouraged the design of this study to develop a country-specific scale measuring the empowerment of Pakistani women building upon the existent evidence and previously developed index in Afghanistan [4] and as was expected, the analyses yielded similar domains as Afghanistan women empowerment index (SWEI-A); however, the property-owning appeared not to be a good fit in the final analyses which left the final model with six domains as explained earlier. This could be explained by the fact that women in Islamic states such as Afghanistan and Pakistan, particularly those from poor families, often received a piece of land or house in form of dowry to consent to marriage; therefore, owning a land or house does not necessarily means that these women are more empowered [50].

Another distinctive feature of our study was measuring the convergence validity of the 6-factor model through cross-examination of emerged domains by four indicators of access to reproductive and maternity care. There are a few studies concerning the impact that women's empowerment could have on access to reproductive and maternity care across the included outcomes in this study among women aged 15–49 years in Pakistan [21–23, 51]; yet the designs did not address the multidimensional aspects of women's empowerment and the outcomes were different in these studies; thus, the findings are either incomparable or inconsistent across studies. For example, Siddique et al., explored the impact that women's empowerment could have on access to ANC among Pakistani women aged 15–49 years using the data from PDHS 2017–18. The results indicated higher access among those with higher education and income, those with managerial positions, those who can make a decision concerning their health care, mobility, and income, and those who are against wife-beating; however, the authors ignored the confounding effect of household’s wealth index‒which we did‒ in examining the effect of different women empowerment indicators on adequate ANC and yet reported it as an independent variable influencing the access to ANC [22]. Similar findings were observed in Asim et al.’s study [23]; however; the authors used the survey-based women's empowerment index (SWPER) which is to some degree similar to our index but developed based on pooled data from several countries in different regions around the globe and includes some indicators such as frequency of reading newspaper, age difference, and education difference that appeared as poor predictors of women empowerment in our study; meanwhile, it failed to take into account the women participation in the labor market which was the strongest predictor of women empowerment in our study [20], and emphasize the importance of a country-specific scale. Another study by Hou and Ma supported the positive effect of women's decision-making on the utilization of reproductive and maternity care services using the data from Pakistan Social and Living Standards Measurement Survey, yet not addressed the impact of other domains of women empowerment that emerged in our study [21]. The study by Herlad et al., found that there is a strong association between women's empowerment and utilization of maternal health care using a researcher-made index including four indicators reflecting upon the woman’s control over personal health and freedom of movement; nonetheless, the index failed to capture the multidimensional nature of women empowerment [51] which we did in designing the index in the present study.

All these being said, we believe that our index is the first scale developed based on data from a nationally representative survey, capturing all the dimensions and embedded domains suggested by existent literature and could reliably reflect upon the women empowerment among married Pakistani women aged 15–49 years in Pakistan. Our survey-based women empowerment index in Pakistan (SWEI-P) not only has important implications for policies and interventions in the country but could also inform the design of future studies and produce comparable results across studies. However, some limitations should be considered in the interpretation of the results. First, the possibility of socially desirable responses due to the self-reported data could lead to biased estimates for included variables. Second, the cultural difference in perception of women's empowerment is not considered in the DHS survey; thus, the answers for some variables, particularly the *attitude toward violence* may be biased. Third, the socioeconomic development of the country may influence the norms and cultural customs over time; therefore, periodical updates are crucial. Lastly; in DHS surveys, most of the questions concerning women empowerment are only asked from married women and single, widows, divorced and separated women are excluded; therefore, the index is only applicable to married women in Pakistan.

## Conclusion

This study was cross-validation of the previously developed index; namely, survey-based women empowerment index in Afghanistan (SWEI-A) that was reformed to represent women empowerment among married Pakistani women. The final index consists of six domains; namely, *labor force participation, attitude toward violence, decision-making, access to healthcare, literacy, age at critical life events,* and could predict the level of women empowerment with high reliability and validity in married Pakistani women aged 15–49 years. There was a significant association between the emerged domains and an at least one of the four indicators of reproductive and maternity care; indicative of a high convergence validity of the index. The survey-based women empowerment index in Pakistan (SWEI-P) proved to be a reliable country-specific index that could measure the empowerment level among marriPed women aged 15–49 years with high accuracy and could inform the design of future policies, interventions, and research recognizing the important indicators of women empowerment in Pakistan and could enhance the comparability of the results across future studies.

## Supplementary Information

**Additional file 1:** **Table S1. **Dimension (D1),Domains (D2), and variables used in describing women’s empowerment. **Table S2.** The frequency and distribution of the included variables, PDHS 2017-18.

## Data Availability

The DHS questionnaire that collected the data in Pakistan's demographic and health survey in 2017–18 could be downloaded from DHS's official website (https://dhsprogram.com/data/available-datasets.cfm). The dataset (PDHS 2017–18) that was used in this study could be available upon a reasonable request and with permission from the DHS website.

## Abbreviations

DHS: Demographic and Health Survey
PDHS: Pakistan Demographic and Health Survey
USAID: United States Agency for International Development
EFA: Explanatory factor analysis
CFA: Confirmatory factor analysis
CFI: Comparative fit index
GDI: Gender Development Index
GEI: Gender Equality Index
GEM: Gender-based Empowerment Measure
KMO: Kaiser–Mayer Olkin
RMSEA: Root mean squared error of approximation
SRMR: Standardized root mean squared residual
PRR: Prevalence rate ratio

## References

[1] Narayan D. Empowerment and Poverty Reduction: A Sourcebook. Washington, DC: World Bank.© World Bank. 2002.

[2] Kabeer N (2005). Gender equality and women's empowerment: a critical analysis of the third millennium development goal 1. Gend Dev.

[3] Duflo E. Gender equality in development. BREAD Policy Paper. 2005;11(4).

[4] Dadras O. Development of a Survey-based Women Empowerment Index for Afghanistan (SWEIA): An Explanatory Analyses of the Afghanistan Demographic Health Survey 2015. In: ResearchSquare, editor. 2022.

[5] Bushra A, Wajiha N (2015). Assessing the Socio-economic Determinants of Women Empowerment in Pakistan. Procedia Soc Behav Sci.

[6] Dadras O, Khampaya T, Nakayama T. Child Marriage, Reproductive Outcomes, and Service Utilization among Young Afghan Women: Findings from a Nationally Representative Survey in Afghanistan. Studies in Family Planning.n/a(n/a). 10.1111/sifp.12207 35736515

[7] Dadras O, Nakayama T, Kihara M, Ono-Kihara M, Dadras F (2022). Intimate partner violence and unmet need for family planning in Afghan women: the implication for policy and practice. Reprod Health.

[8] Charmes J, Wieringa S (2003). Measuring women's empowerment: an assessment of the gender-related development index and the gender empowerment measure. J Hum Dev.

[9] Ahmed S, Creanga AA, Gillespie DG, Tsui AO (2010). Economic status, education and empowerment: implications for maternal health service utilization in developing countries. PLoS ONE.

[10] Lopez-Claros A, Zahidi S, mondial Fé, editors. Women's empowerment: Measuring the global gender gap2005: World Economic Forum Geneva.

[11] Yaya S, Uthman OA, Ekholuenetale M, Bishwajit G (2018). Women empowerment as an enabling factor of contraceptive use in sub-Saharan Africa: a multilevel analysis of cross-sectional surveys of 32 countries. Reprod Health.

[12] Tadesse M, Teklie H, Yazew G, Gebreselassie T. Women’s empowerment as a determinant of contraceptive use in Ethiopia further analysis of the 2011 Ethiopia demographic and health survey DHS Further Analysis Reports 2013 82

[13] Msuya SE, Adinan J, Mosha N. Intimate partner violence and empowerment among women in Tanzania: Prevalence and effect on utilization of reproductive and maternal health services: ICF International; 2014.

[14] Gupta  MD (1995). Life course perspectives on Women's autonomy and health outcomes. Am Anthropol.

[15] Huis MA, Hansen N, Otten S, Lensink R (2017). A three-dimensional model of women’s empowerment: implications in the field of microfinance and future directions. Front Psychol.

[16] Miedema SS, Haardörfer R, Girard AW, Yount KM (2018). Women’s empowerment in East Africa: development of a cross-country comparable measure. World Dev.

[17] Permanyer I (2013). A critical assessment of the UNDP’s gender inequality index. Fem Econ.

[18] Cueva BH (2006). What is missing in measures of women's empowerment?. J Hum Dev.

[19] Dadi D, Bogale D, Minda Z, Megersa S (2020). Decision-making power of married women on family planning use and associated factors in Dinsho Woreda, South East Ethiopia. Open Access J Contracept.

[20] Ewerling F, Lynch JW, Victora CG, van Eerdewijk A, Tyszler M, Barros AJ (2017). The SWPER index for women's empowerment in Africa: development and validation of an index based on survey data. Lancet Glob Health.

[21] Hou XAM, Ning. Empowering women: The effect of women's decision-making power on reproductive health services uptake: Evidence from Pakistan. 10.1093/heapol/czs042 22522771

[22] Siddique K, Malik R, Batool I, Usman A, Bin NS (2022). Women’s Empowerment and Antenatal Care Utilization in Pakistan. J Int Women's Stud.

[23] Asim M, Hameed W, Saleem S (2022). Do empowered women receive better quality antenatal care in Pakistan? An analysis of demographic and health survey data. PLoS ONE.

[24] Abbas S, Isaac N, Zia M, Zakar R, Fischer F (2021). Determinants of women’s empowerment in Pakistan: evidence from Demographic and Health Surveys, 2012–13 and 2017–18. BMC Public Health.

[25] Studies NIoP, ICF. Pakistan demographic and health survey 2017–18. NIPS/Pakistan and ICF Islamabad, Pakistan; 2019.

[26] Kabeer N (1999). Resources, agency, achievements: Reflections on the measurement of women's empowerment. Dev Chang.

[27] Malhotra A, Schuler SR, Boender C, editors. Measuring women’s empowerment as a variable in international development. background paper prepared for the World Bank Workshop on Poverty and Gender: New Perspectives; 2002: The World Bank Washington, DC.

[28] Asaolu IO, Alaofè H, Gunn JK, Adu AK, Monroy AJ, Ehiri JE (2018). Measuring women's Empowerment in sub-Saharan Africa: exploratory and confirmatory factor analyses of the demographic and health surveys. Front Psychol.

[29] Phan L (2016). Measuring Women’s Empowerment at Household Level Using DHS Data of Four Southeast Asian Countries. Soc Indic Res.

[30] Worthington RL, Whittaker TA (2006). Scale development research: a content analysis and recommendations for best practices. Couns Psychol.

[31] Cabrera-Nguyen P (2010). Author guidelines for reporting scale development and validation results in the Journal of the Society for Social Work and Research. J Soc Soc Work Res.

[32] Gaskin CJ, Happell B (2014). On exploratory factor analysis: a review of recent evidence, an assessment of current practice, and recommendations for future use. Int J Nurs Stud.

[33] Stevens JP. Applied Multivariate Statistics for the Social Sciences. 5th Edition ed. New York: Routledge; 2009.

[34] Bland JM, Altman DG (1997). Cronbach's alpha Bmj.

[35] Bland JM, Altman DG (2002). Statistics notes: validating scales and indexes. BMJ.

[36] Lyne J, Renwick L, Grant T, Kinsella A, McCarthy P, Malone K (2013). Scale for the assessment of negative symptoms structure in first episode psychosis. Psychiatry Res.

[37] Raine A, Wong KK, Liu J (2021). The Schizotypal Personality Questionnaire for Children (SPQ-C): Factor Structure, Child Abuse, and Family History of Schizotypy. Schizophr Bull.

[38] Schreiber JB, Nora A, Stage FK, Barlow EA, King J (2006). Reporting structural equation modeling and confirmatory factor analysis results: a review. J Educ Res.

[39] Brown TA. Confirmatory factor analysis for applied research: Guilford publications; 2015.

[40] Mumtaz S. Women's empowerment and maternal and child healthcare services utilization in muslim countries in South Asia. Management. 2018.

[41] Tarekegn SM, Lieberman LS, Giedraitis V (2014). Determinants of maternal health service utilization in Ethiopia: analysis of the 2011 Ethiopian Demographic and Health Survey. BMC Pregnancy Childbirth.

[42] Barros AJ, Hirakata VN (2003). Alternatives for logistic regression in cross-sectional studies: an empirical comparison of models that directly estimate the prevalence ratio. BMC Med Res Methodol.

[43] Wendt A, Santos TM, Cata-Preta BO, Costa JC, Mengistu T, Hogan DR (2022). Children of more empowered women are less likely to be left without vaccination in low- and middle-income countries: a global analysis of 50 DHS surveys. J Glob Health.

[44] Kline R. Principles and practice of structural equation modeling: Guilford publications 2015.

[45] Carter R (2006). Solutions for missing data in structural equation modeling. Res Pract  Assess.

[46] Hameed W, Azmat SK, Ali M, Sheikh MI, Abbas G, Temmerman M (2014). Women's empowerment and contraceptive use: the role of independent versus couples' decision-making, from a lower middle income country perspective. PLoS ONE.

[47] Studies. AIoA, Studies. AIoP. Afghanistan And Pakistan: Cultural Heritage and Current Reality. Istanbul, Turkey: The Hollings Center for International Dialogue; 2005.

[48] Dadras O, Nakayama T, Kihara M, Ono-Kihara M, Seyedalinaghi S, Dadras F (2021). The prevalence and associated factors of adverse pregnancy outcomes among Afghan women in Iran; Findings from community-based survey. PLoS ONE.

[49] Yount KM, Crandall A, Cheong YF (2018). Women's age at first marriage and long-term economic empowerment in EGYPT. World Dev.

[50] Makino M (2019). Marriage, dowry, and women’s status in rural Punjab. Pakistan J Popul Econ.

[51] Hearld KR, Anderson JL, Budhwani H (2018). Examining the relationship between individual characteristics, community-level traits, multidimensional empowerment, and maternal health care utilization in the Islamic Republic of Pakistan. Matern Child Health J.

