# Supplementary material for: Women empowerment and access to maternity and reproductive healthcare in Pakistan: cross-validation of a Survey-based Index in Afghanistan (SWEI-A)
Source: BMC Womens Health. 2022 Nov 16;22:453. doi: 10.1186/s12905-022-02031-2 (PMC9670496; doi:10.1186/s12905-022-02031-2)
Supplement: Supplementary file 1 — Additional file 1: Table S1. Dimension (D1),Domains (D2), and variables used in describing women’s empowerment. Table S2. The frequency and distribution of the included variables, PDHS 2017-18. [file 12905_2022_2031_MOESM1_ESM.docx]

| **Table S1.** Dimension (D1), Domains (D2), and variables used in describing women’s empowerment. | | | | | |
| --- | --- | --- | --- | --- | --- |
| **D1** | **D2** | **Indicator** | **Questions** | **DHS response categories** | **Recode used in analysis** |
| **Economic** | Labor Force Participation | Occupation | Type of work | Not working=0; professional/technical/managerial=1; clerical=2; sales=3; agricultural - self employed=4;  services=7; skilled manual=8  unskilled manual=9 | Not working=0; non-skilled ( unskilled manual/clerical/ services)=1; agriculture self-employed=2; skilled worker ( professional/technical/managerial/ skilled manual)=3 |
|  |  | Earning | Type of earnings from respondent's work | Not paid=0 Cash only=1 Cash and in-kind=2 In-kind only=1 | Not working=0, Not-paid=1, In-kind only=2, Cash and in-kind only=3, Cash only=4 |
|  |  | Seasonality | Respondent employed all year/seasonal | All year=1, Seasonal=2, Occasional=3 | Not working=0, Occasional=1, Seasonal=2, All year=3 |
|  |  | Income Ratio | Respondent earns more than husband/partner | More than him=1, Less than him=2, About the same=3 Husband/partner has no income=4 Don’t know=8 | Not working=0, Husband/partner has no income/ don’t know/Less than him=1, About the same=2, More than him=3 |
|  |  | Work autonomy | Who do you work for? | Family member=1, Someone else=2, self-employed=3 | Not working=0, Family member=1, Someone else=2, Self-employed=3 |
|  | Property-owning | Land ownership | Owns land alone or jointly | Does not own=0, Alone only=1, Jointly only=2, Both alone and jointly=3 | Does not own=0, Jointly only=1, Alone only=2, Both alone and jointly=3 |
|  |  | House ownership | Owns a house alone or jointly | Does not own=0, Alone only=1, Jointly only=2, Both alone and jointly=3 | Does not own=0, Jointly only=1, Alone only=2, Both alone and jointly=3 |
| **Socio-Cultural** | Household Decision-making | Women's health | Person who usually decides on respondent's healthcare | Respondent alone=1  Respondent and husband/partner=2  Husband/partner alone=4  Someone else=5  Other=6 | Husband/partner alone/Someone else/Other=0, Respondent and husband/partner=1,  Respondent alone=2 |
|  |  | Large household purchases | Person who usually decides on large household purchases |  |  |
|  |  | Visiting relatives/family | Person who usually decides on visits to family or relatives |  |  |
|  | Attitudes towards violence | Goes out without telling husband | Beating justified if wife goes out without telling husband | No=0,  Yes=1,  Don't know=8 | No=1,  Yes/ Don't know =0 |
|  |  | Neglects children | Beating justified if wife neglects the children |  |  |
|  |  | Argues with husband | Beating justified if wife argues with husband |  |  |
|  |  | Refuses sex | Beating justified if wife refuses to have sex with husband |  |  |
|  |  | Burns food | Beating justified if wife burns the food |  |  |
|  | Age at critical life event | Age at first birth | Age of respondent at first birth | Age in years | No change |
|  |  | Age at cohabitation | Age at first cohabitation | Age in years | No change |
| **Education** | Literacy | Literacy | Reading abilities | Cannot read at all=0, Able to read only parts of sentence=1, Able to read whole sentence=2 | No change |
|  |  |  | Frequency of reading newspaper | Not at all=0, Less than once a week=1, At least once a week=2 | No change |
|  |  | Educational level | Highest educational level | No education=0; Primary=1; Secondary=2; Higher=3 | No change |
| **Health** | Negotiating sex | Can ask partner to use condom | Respondent can ask partner to use a condom | No=0,  Yes=1  Don't know/not sure/depends=8 | No/ Don't know/not sure/depends=0  Yes=1 |
|  |  | Can say no to sex | Respondent can refuse sex |  |  |
|  | Access to Healthcare | Permission | Getting permission to go | Not a big problem=2; Big problem=1 | No change |
|  |  | Money | Getting money needed for treatment |  |  |
|  |  | Distance | Distance to health facility |  |  |
|  |  | Going Alone | Not wanting to go alone |  |  |

| **Table S2. The frequency and distribution of the included variables, PDHS 2017-18** | | | | |
| --- | --- | --- | --- | --- |
| **D2** | **Indicator** | **Questions** | **Recode used in analysis** | **N (weighted%)** |
| Labor Force Participation | Occupation | Type of work | Not working | 12389 (85.43) |
|  |  |  | non-skilled ( unskilled | 368 (2.52) |
|  |  |  | agriculture self-employed | 470 (3.21) |
|  |  |  | skilled worker | 1268 (8.74) |
|  | Earning | Type of earnings | Not working | 12389 (85.43) |
|  |  |  | Not-paid | 208 (2.47) |
|  |  |  | In-kind only | 35 (0.44) |
|  |  |  | Cash and in-kind only | 67 (0.80) |
|  |  |  | Cash only | 1803 (10.86) |
|  | Seasonality | employed all year/seasonal | Not working | 12389 (85.43) |
|  |  |  | Occasional | 223 (1.55) |
|  |  |  | Seasonal | 404 (2.79) |
|  |  |  | All year | 1485 (10.23) |
|  | Income Ratio | Respondent earns compared to husband | Not working | 12389 (85.43) |
|  |  |  | Husband has no income/ don’t know/Less than him | 1455 (11.20) |
|  |  |  | About the same | 201 (1.12) |
|  |  |  | More than him | 210 (1.15) |
|  | Work autonomy | Who do you work for? | Not working | 12389 (85.43) |
|  |  |  | Family member | 595 (4.10) |
|  |  |  | Someone else | 934 (6.44) |
|  |  |  | Self-employed | 583 (4.03) |
| Property-owning | Land ownership | Owns land alone or jointly | Does not own | 14226 (98.12) |
|  |  |  | Jointly only | 115 (0.79) |
|  |  |  | Alone only | 139 (0.96) |
|  |  |  | Both alone and jointly | 18 (0.12) |
|  | House ownership | Owns a house alone or jointly | Does not own | 14115 (97.36) |
|  |  |  | Jointly only | 173 (1.19) |
|  |  |  | Alone only | 189 (1.30) |
|  |  |  | Both alone and jointly | 21 (0.14) |
| Household Decision-making | Women's health | who usually decides on respondent's healthcare | Husband/partner alone/Someone else/Other | 7507 (51.77) |
|  |  |  | Respondent and husband/partner | 5594 (38.58) |
|  |  |  | Respondent alone | 1399 (9.65) |
|  | Large household purchases | Person who usually decides on large household purchases | Husband/partner alone/Someone else/Other | 8488 (58.54) |
|  |  |  | Respondent and husband/partner | 5151 (35.52) |
|  |  |  | Respondent alone | 861 (5.94) |
|  | Visiting relatives/family | Person who usually decides on visits to family or relatives | Husband/partner alone/Someone else/Other | 7767 (53.57) |
|  |  |  | Respondent and husband/partner | 5306 (36.59) |
|  |  |  | Respondent alone | 1427 (9.84) |
| Attitudes towards violence | Goes out without telling husband | Beating justified if wife goes out without telling husband | No | 8900 (61.38) |
|  |  |  | Yes/ Don’t know | 5600 (38.62) |
|  | Neglects children | Beating justified if wife neglects the children | No | 9782(67.46) |
|  |  |  | Yes/ Don’t know | 4718 (32.54) |
|  | Argues with husband | Beating justified if wife argues with husband | No | 8931 (61.59) |
|  |  |  | Yes/ Don’t know | 5569 (38.41) |
|  | Refuses sex | Beating justified if wife refuses to have sex with husband | No | 9549 (5.86) |
|  |  |  | Yes/ Don’t know | 4950 (34.14) |
|  | Burns food | Beating justified if wife burns the food | No | 11385 (78.52) |
|  |  |  | Yes/ Don’t know | 3115 (21.48) |
| Age at critical life event | Age at first birth (year) | Age of respondent at first birth (mean/SD) |  | 21.27 (0.90) |
|  | Age at cohabitation (year) | Age at first cohabitation(mean/SD) |  | 19.44 (0.93) |
| Literacy | Literacy | Reading abilities | Cannot read at all | 7454 (51.41) |
|  |  |  | Able to read only parts of sentence | 833 (5.74) |
|  |  |  | Able to read whole sentence | 6206 (42.85) |
|  |  | Frequency of reading newspaper | Not at all | 12006 (82.85) |
|  |  |  | Less than once a week | 1599 (11.03) |
|  |  |  | At least once a week | 887 (6.12) |
|  | Educational level | Highest educational level | No educatio | 7313 (50.43) |
|  |  |  | Primary | 2022 (13.94) |
|  |  |  | Secondary | 3023 (20.85) |
|  |  |  | Higher | 2.144 (14.78) |
| Negotiating sex | Can ask partner to use condom | Respondent can ask partner to use a condom | No/ Don't know/not sure/depends | 7984 (55.07) |
|  |  |  | Yes | 6514 (89.33) |
|  | Can say no to sex | Respondent can refuse sex | No/ Don't know/not sure/depends | 6514 (44.03) |
|  |  |  | Yes | 7983 (55.07) |
| Access to Healthcare | Permission | Getting permission to go | Not a big problem | 10962 (75.63) |
|  |  |  | Big problem | 3532 (24.37) |
|  | Money | Getting money needed for treatment | Not a big problem | 9546 (65.86) |
|  |  |  | Big problem | 4948 (34.14) |
|  | Distance | Distance to health facility | Not a big problem | 7674 (52.95) |
|  |  |  | Big problem | 6819 (47.05) |
|  | Going Alone | Not wanting to go alone | Not a big problem | 5475 (37.77) |
|  |  |  | Big problem | 9019 (62.23) |
